# Distinct N and C Cross-Feeding Networks in a Synthetic Mouse Gut Consortium

**DOI:** 10.1128/msystems.01484-21

**Published:** 2022-03-31

**Authors:** Pau Pérez Escriva, Tobias Fuhrer, Uwe Sauer

**Affiliations:** a Institute of Molecular Systems Biology, D-BIOL, ETH Zurich, Zurich, Switzerland; b Systems Biology Graduate School, Zurich, Switzerland; Oregon State University

**Keywords:** food web, metabolic interactions, metabolism, metabolomics, microbial communities

## Abstract

The complex interactions between the gut microbiome and host or pathogen colonization resistance cannot be understood solely from community composition. Missing are causal relationships, such as metabolic interactions among species, to better understand what shapes the microbiome. Here, we focused on metabolic niches generated and occupied by the Oligo-Mouse-Microbiota (OMM) consortium, a synthetic community composed of 12 members that is increasingly used as a model for the mouse gut microbiome. Combining monocultures and spent medium experiments with untargeted metabolomics revealed broad metabolic diversity in the consortium, constituting a dense cross-feeding network with more than 100 pairwise interactions. Quantitative analysis of the cross-feeding network revealed distinct C and N food webs, highlighting the two *Bacteroidetes* members Bacteroides caecimuris and Muribaculum intestinale as primary suppliers of carbon and a more diverse group as nitrogen providers. Cross-fed metabolites were mainly carboxylic acids, amino acids, and the so far not reported nucleobases. In particular, the dicarboxylic acids malate and fumarate provided a strong physiological benefit to consumers, presumably used in anaerobic respiration. Isotopic tracer experiments validated the fate of a subset of cross-fed metabolites, such as the conversion of the most abundant cross-fed compound succinate to butyrate. Thus, we show that this consortium is tailored to produce the anti-inflammatory metabolite butyrate. Overall, we provide evidence for metabolic niches generated and occupied by OMM members that lays a metabolic foundation to facilitate an understanding of the more complex *in vivo* behavior of this consortium in the mouse gut.

**IMPORTANCE** This article maps out the cross-feeding network among 10 members of a synthetic consortium that is increasingly used as the model mouse gut microbiota. Combining metabolomics with *in vitro* cultivations, two dense networks of carbon and nitrogen exchange are described. The vast majority of the ∼100 interactions are synergistic in nature, in several cases providing distinct physiological benefits to the recipient species. These networks lay the groundwork toward understanding gut community dynamics and host-gut microbe interactions.

## INTRODUCTION

The mammalian gut microbiome is a complex community with thousands of bacterial species (1) that affects many facets of host physiology, ranging from metabolism and the development of the immune system to protection against pathogens (2). Extensive sequencing efforts categorized gut inhabitants and their genetic repertoire (3), but fecal microbiome composition alone does not reveal the spatial and dynamic interactions between its members and with the host. These species interactions determine the succession, stability, and resilience of a community (1) and are the basis of causal relationships between microbiome composition and host physiology. Beyond correlative sequencing efforts, contemporary assessment of causal links is restricted to individual species (4) or genes (5). Understanding more complex behavior such as pathogen colonization, however, requires considering communities at large, which is hampered by technical limitations for *in vivo* studies. Recent *in vitro* studies demonstrated that elucidating the nature of pairwise interactions between community members can be used to predict the assembly and dynamic behavior of a community (6–8). Such pairwise interactions can be neutral, negative for both partners (competition) or one partner (ammensalism), or positive for both partners (mutualism) or one partner (commensalism). The underlying basis may be physical (9, 10), quorum sensing (11), toxins, competition for nutrients (12), or metabolic cross-feeding.

To reduce the daunting complexity of natural systems, model communities of the gut microbiome with a defined species composition have been used to colonize germfree animals (13). The primary focus of such models is to investigate complex phenotypes such as the interplay between the microbiome and the host immune system or pathogen colonization resistance (14). Recently, the Oligo-Mouse-Microbiota (OMM) consortium was introduced as a model for the mouse gut microbiome to study colonization resistance (15). Composed of 12 natural murine isolates representing the five main gut phyla, it confers higher colonization resistance toward the pathogen Salmonella enterica than the classical 8-species altered Schaedler flora consortium (15). Importantly, it is stable over time and reproducibly maintained in different animal facilities, rendering it an attractive model for the gut microbiome (16). Although developed only recently, the OMM consortium has already helped to deepen our understanding of colonization resistance (15), inflammation (17), and the development of the immune system (18).

Generally, metabolic activities and interactions between species remain largely unexplored, even for these relatively simple, synthetic consortia. Analyzing extracellular metabolic changes upon growth in culture supernatants or in cocultures revealed parts of a food web within the altered Schaedler flora consortium (19). A first physiological characterization of microbial interactions within the OMM consortium reported primarily exploitative and interference competition during *in vitro* growth on culture supernatants (20). From exometabolome changes in these cultures, those authors found the substrate depletion profiles to correlate with growth inhibition, identified several species-specific substrates and products, and singled out Enterococcus faecalis as the major determinant of community composition (20), although it is only a low-abundance member of the healthy gut microbiome (21). Actual cross-feeding of metabolites was hypothesized between Clostridium innocuum and E. faecalis.

Here, we focus on unraveling cross-feeding systematically between all OMM species and ask whether such metabolic interactions could also be beneficial in nature rather than the reported competitive interactions, thereby contributing to community stability. Dynamic exometabolome changes during growth in complex medium and in culture supernatants of other consortium members revealed broad metabolic diversity among the OMM members that gave rise to a dense cross-feeding network with more than 100 pairwise interactions, where the most abundant *in vivo* members were the main providers. We unraveled two distinct food webs of carbon and nitrogen sources that highlight *Bacteroidetes* as primary suppliers of C and *Firmicutes* as well as the *Bacteroidetes* member Muribaculum intestinale as providers of N-containing compounds. The fate of several relevant cross-fed compounds was experimentally validated by isotopic tracing, allowing us to understand their metabolic fate within the community. We thus provide evidence for key metabolic niches that are generated and occupied by members of the OMM consortium and the individual roles of each member within it.

## RESULTS

### Physiological and metabolic diversity within the OMM consortium.

**FIG 1:**
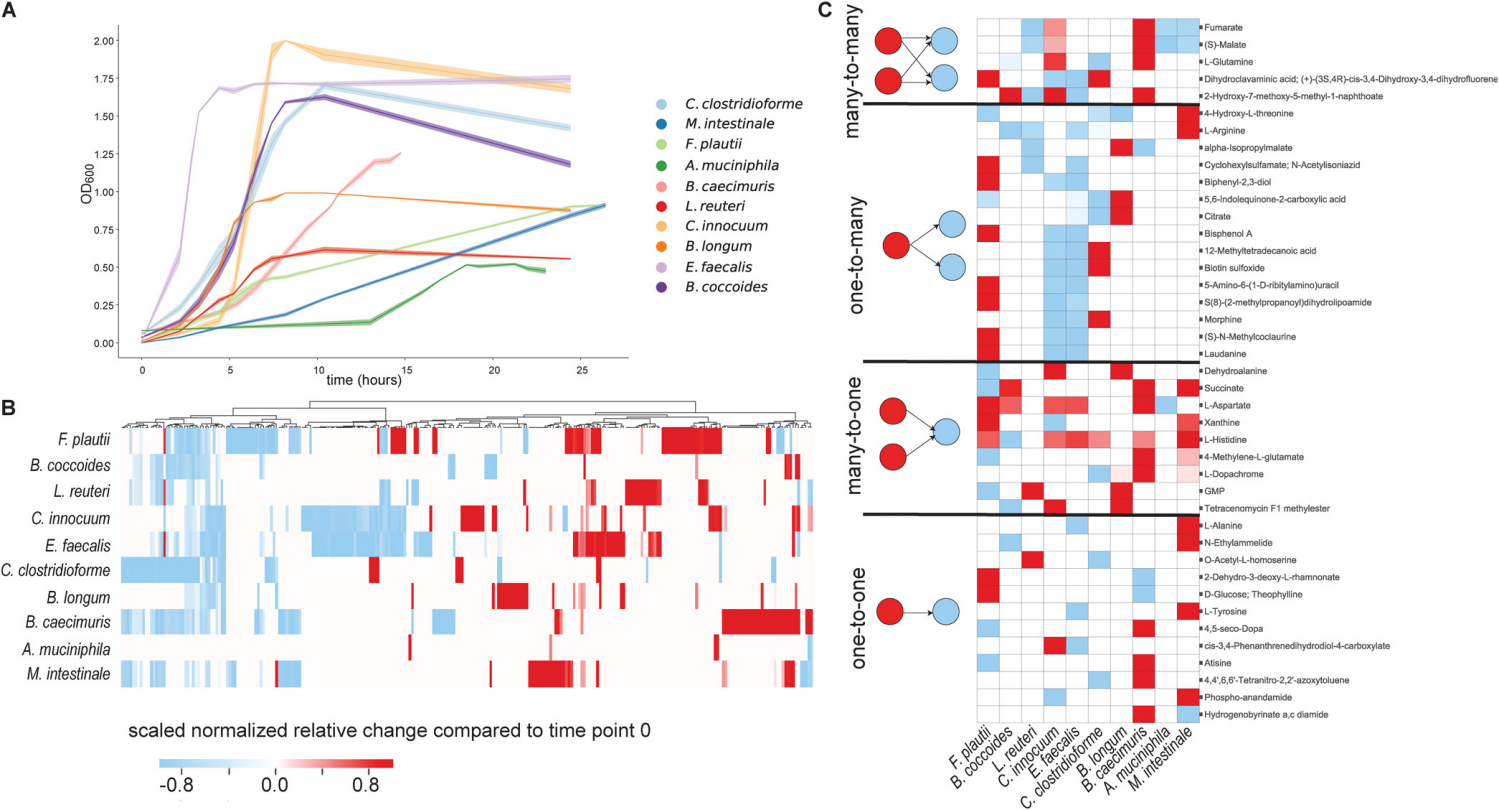
Exometabolome dynamics of OMM species in mBHI medium. (A) Growth curves of 10 OMM species in mBHI medium. Shaded areas indicate the standard deviations from the means (*n* = 3 to 4 replicates). (B) Metabolic footprint heat map of all 10 OMM species during growth in mBHI medium. Secretion is indicated in red, and consumption is in blue. Intensities are scaled to ±1 by dividing each metabolite by the maximum observed change in abundance in all species. Hierarchical clustering was performed for metabolites, using Euclidian distances and centroid linkage. (C) Heat map describing the different types of potential interactions extrapolated from the consumption and secretion profiles in panel B.

To characterize physiology and the secretion of metabolic products, each member of the OMM consortium was grown anaerobically in brain heart infusion (BHI) broth supplemented with hemin, the vitamin K precursor menadione, and mucin as the key constituent of the gut mucus (modified BHI [mBHI] medium) (22). Representing <5% of the fecal bacterial load of mice carrying the OMM consortium (15), the two minor constituents Turicimonas muris and Acutalibacter muris did not grow under these conditions. The other 10 species achieved their maximum optical densities (ODs) at 600 nm (OD_600_) in mBHI medium within 25 h (Fig. 1A). As the major constituents of the fecal community, with up to 50% (21), the *Bacteroidetes* phylum representatives Bacteroides caecimuris and M. intestinale exhibited similar lag phases of 4 to 8 h and maximum ODs, but *M. intestinale* grew substantially slower (Table 1). The *Firmicutes* had shorter lag phases and displayed broader ranges of maximum ODs and specific growth rates (Fig. 1A and Table 1). The mucus-degrading constituent Akkermansia muciniphila grew only to a low maximum OD and did not grow in the absence of mucin (see Table S1A in the supplemental material), suggesting that mucin is its main carbon source, as shown previously (23).

**TABLE 1:** Physiological properties of the OMM members in mBHI medium^*a*^

Phylum and species	Code	Max OD	Mean μ ± SD
*Firmicutes*			
Flavonifractor plautii	YL31	0.92	0.49 ± 0.08
Blautia coccoides	YL58	1.73	0.6 ± 0.07
Lactobacillus reuteri	I49	0.62	0.45 ± 0.04
Clostridium innocuum	I46	1.98	1.34 ± 0.13
Enterococcus faecalis	KB1	1.78	2.5 ± 0.01
Acutalibacter muris	KB18	—	—
Clostridium clostridioforme	YL32	1.74	0.54 ± 0.02

*Actinobacteria*			
Bifidobacterium longum	YL2	1.01	0.91 ± 0.04

*Proteobacteria*			
Turicimonas muris	YL45	—	—

*Bacteroidetes*			
Bacteroides caecimuris	I48	1.18	0.47 ± 0.02
Muribaculum intestinale	YL27	0.93	0.39 ± 0.03

*Verrucomicrobia*			
Akkermansia muciniphila	YL44	0.32	0.09 ± 0.01

a Cultures were grown in 10 mL of mBHI medium under anaerobic conditions in Hungate tubes. Dashes represent not determined data; Code represents the strain designation.

10.1128/msystems.01484-21.7 TABLE S1 (A) Growth of *A. muciniphila* over time in mBHI medium with or without mucin. (B) Relative maximum OD obtained in spent medium experiments. (C) Fresh and spent medium experiment sampling metadata. (D) OMM genome-based inferred metabolic potential. Download Table S1, XLSX file, 0.08 MB. Copyright © 2022 Pérez Escriva et al. 2022 Pérez Escriva et al. https://creativecommons.org/licenses/by/4.0/ This is an open-access article distributed under the terms of the Creative Commons Attribution 4.0 International license.

To characterize the metabolism of each species, we determined consumed and secreted metabolites by untargeted flow injection analysis-time of flight mass spectrometry (FIA-TOFMS) (24). Specifically, 3 to 4 biological replicates were grown per species, and 8 to 10 aliquots of the culture supernatants were sampled throughout the growth phase of each and measured by FIA-TOFMS. A total of 713 detected ions could be annotated to metabolites based on accurate mass, assuming single deprotonation, and 268 had changing time profiles in at least 1 of the 10 investigated species (Table S2A). Additionally, amino acids and short-chain fatty acids (SCFAs) were quantified by a targeted liquid chromatography-mass spectrometry (LC-MS) method. The 29 metabolites consumed by at least half of the species were primarily organic acids such as pyruvate and 2-oxobutanoate; amino acid derivatives such as 4-phospho-l-aspartate, *N*-succinyl-l-citrulline, and 5-hydroxy-l-tryptophan; and a few micronutrients such as ascorbate (Table S2A and Fig. S1A and C).

10.1128/msystems.01484-21.1 FIG S1 (A) Metabolites consumed (blue) by more than half of the OMM members. Metabolites that were not detected as being abundant in fresh mBHI medium are shown in boldface type. Intensities are scaled to ±1 by dividing each metabolite by the maximum observed change in abundance in all species. (B) Metabolites that were uniquely secreted by one member of the consortium. Metabolites that were not detected as being abundant in fresh mBHI medium are shown in boldface type. Alternative annotations within the mass tolerance of 3 mDa are indicated where present. (C) Upset plot of metabolites consumed by the OMM species. (D) Upset plot of metabolites produced by the OMM species. Download FIG S1, TIF file, 2.4 MB. Copyright © 2022 Pérez Escriva et al. 2022 Pérez Escriva et al. https://creativecommons.org/licenses/by/4.0/ This is an open-access article distributed under the terms of the Creative Commons Attribution 4.0 International license.

10.1128/msystems.01484-21.8 TABLE S2 (A) Secreted and consumed metabolites from OMM members when grown in mBHI medium. (B) Inferred cross-feeding interactions from spent medium cultures. (C) Metabolic consumption secretion ratios for OMM members. (D) Metabolic class of cross-fed metabolites. Download Table S2, XLSX file, 0.06 MB. Copyright © 2022 Pérez Escriva et al. 2022 Pérez Escriva et al. https://creativecommons.org/licenses/by/4.0/ This is an open-access article distributed under the terms of the Creative Commons Attribution 4.0 International license.

Despite their abundance in mBHI medium, surprisingly, none of the amino acids was consumed by the majority of the members (Fig. S2). Besides serving as precursors for biomass, amino acids can also be used as an energy source, for example, in Stickland fermentations, where one amino acid serves as an electron donor and another serves as an acceptor by forming carboxylic acids (25). Many gut microbes are capable of Stickland reactions that may play a role in cross-feeding in the gut (26). Several OMM members have the capacity to degrade arginine, alanine, glycine, leucine, or aspartate by Stickland fermentation (20). The observed degradation of aspartate by A. muciniphila and alanine by E. faecalis might thus be explained by Stickland fermentation (Fig. S2). Although all OMM members could potentially degrade arginine via Stickland reduction, only four members consumed it under our conditions (Fig. S2). Other types of amino acid degradation were, for example, seen for lysine and histidine that were consumed by Flavonifractor plautii and Blautia coccoides, respectively, the only OMM members able to degrade these amino acids (Fig. S2 and Table S1D) (27). Lysine degradation might be relevant *in vivo* for this consortium since its degradation by F. plautii yields two of the three classical short-chain fatty acids, acetate and butyrate. Although nine species encode l-serine dehydratase orthologs that can catalyze the degradation of serine to pyruvate, only the fast-growing species E. faecalis and, to a lesser extent, Clostridium clostridioforme and *F. plautii* consumed serine in large amounts (Fig. S2 and Table S1D).

10.1128/msystems.01484-21.2 FIG S2 Consumption (blue) and secretion (red) of relevant metabolite classes by OMM species. Consumption or secretion was inferred by comparing late-stationary-phase concentrations in spent media to concentrations in fresh mBHI medium, scaled to ±1 by dividing the relative concentration of each metabolite by the maximum observed change in abundance in all species. (A) Nucleic acid profiles determined by untargeted metabolomics analysis with FIA-MS. (B) Short-chain fatty acid profiles determined by 3-NPH derivatization (71) coupled with targeted metabolomics. (C) Vitamin and cofactor profiles determined by untargeted metabolomics analysis with FIA-MS. (D) Amino acid profile determined by HILIC–LC-MS. Asterisks indicate species with the ability to degrade an amino acid via Stickland fermentation that consumed the respective amino acid. Download FIG S2, EPS file, 2.0 MB. Copyright © 2022 Pérez Escriva et al. 2022 Pérez Escriva et al. https://creativecommons.org/licenses/by/4.0/ This is an open-access article distributed under the terms of the Creative Commons Attribution 4.0 International license.

Typical end products of fermentation such as short-chain fatty acids (28) and amino acid derivatives (29) were produced by some species. In particular, acetate was secreted by most consortium members (Fig. S2). The short-chain fatty acid butyrate that is used by enterocytes as an energy source (30) was produced by C. clostridioforme, *F. plautii*, and C. innocuum (Fig. S2), where the latter two have the genetic repertoire for its production from sugars (27). Propionate was secreted by *A. muciniphila* and the two *Bacteroidetes* members with succinate pathway genes, the only propionate production pathway from carbohydrates known for this phylum (31). Accumulation of amino acids, most likely from peptide digestion (32), was seen for the two *Bacteroidetes* members and several *Clostridia* (Fig. S2). Amino acid fermentation products such as isopropyl-malate, 4-aminobutanoate, 4-methyl-oxopentanoate, and 3-methyl-2-oxobutanoic acid were secreted by *C. innocuum*, Bifidobacterium longum, and *M. intestinale* (Table S2A). While many metabolites were secreted by several species, none was secreted by all (Fig. 1B). The large numbers of metabolites (90 [33% of all changing metabolites]) secreted by only one species and several taxon-specific metabolites suggest broad metabolic diversity (Fig. S1B and D). With 52 secreted metabolites (19% of all the metabolites detected to change over time), *F. plautii* was not only the main producer in the consortium but also the unique source of 23 metabolites (Fig. S1D).

To start mapping out the metabolic interaction network from the consumption and secretion patterns in monocultures, we selected for metabolites that were secreted by at least one member of the consortium and consumed by one or more members. From the 268 compounds with dynamic profiles, we predict 15 as one-to-many, 12 as one-to-one, 9 as many-to-one, and 5 as many-to-many cross-feeding interactions (Fig. 1C). In total, 41 metabolites were secreted by at least one member and consumed by at least one other and, hence, are potentially cross-fed.

### The OMM metabolic food web is highly connected.

**FIG 2:**
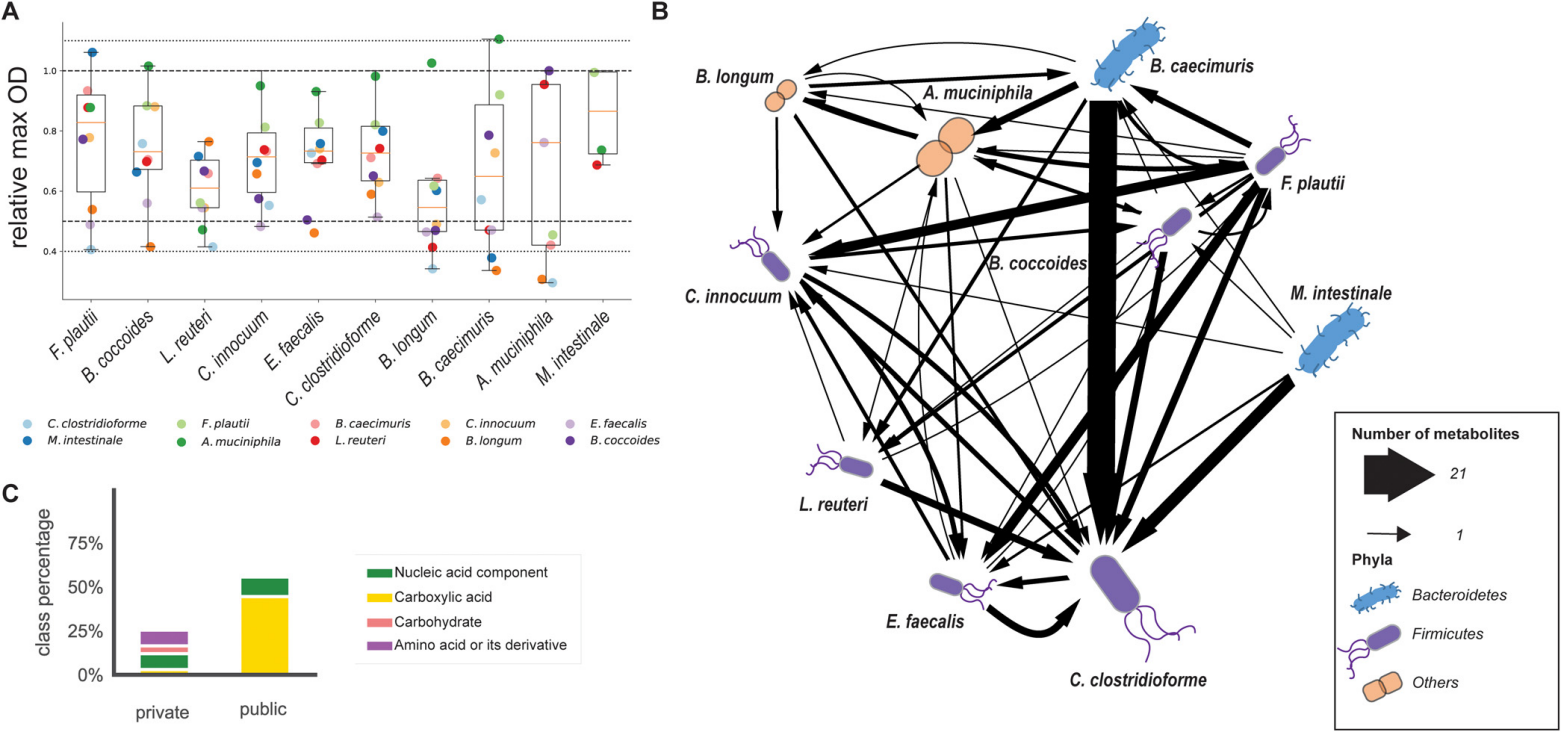
Metabolite cross-feeding among OMM members in mBHI spent medium. (A) Maximum OD of OMM species during growth in a spent medium mixture of 50% culture supernatant and 50% mBHI medium. Data points are the means from duplicate measurements (see Table S1B in the supplemental material). The species origin of the culture supernatant is indicated by the color of the points. The relative maximum OD achieved by each of the other species is given on the *y* axis, relative to the maximum OD achieved in fresh mBHI medium. Relative maximum ODs of 1.1, 1, 0.5, and 0.4 are indicated by the dotted lines. (B) Metabolite interaction network of OMM species inferred from spent medium experiments. Metabolites secreted by a producer in fresh mBHI medium and consumed in spent media by a second species were classified as cross-fed. Consumed metabolites in spent medium experiments were identified by filtering all decreasing annotated ions, based on either a significant correlation with the culture OD over time (Pearson correlation coefficient of less than −0.7; *P* value of <0.05) or a significant goodness of linear or exponential fit (*R*^2^ of >0.7; *P* value of <0.05). Bacteria that represent more than 10% of the community are shown in a larger size in a study by Yilmaz et al. (21). (C) Relative metabolite class distributions of public and private cross-fed compounds. Percentages were calculated from the total numbers of metabolites within a class divided by the total number of metabolites, including the ones without a specific class associated.

To obtain more direct evidence for cross-feeding and to capture interactions through secreted metabolites that were not already present in mBHI medium, we performed systematic pairwise cultivation experiments. For this purpose, cell-free culture supernatants of all 10 species were harvested at the maximum OD in mBHI medium. These supernatants (i.e., spent media) were mixed at a ratio of 1 to 1 with fresh mBHI medium to ensure some bacterial growth and inoculated with each of the other nine species in duplicates. To assess the influence of spent media on consumers, we compared the maximum OD obtained in the spent medium to the one obtained in undiluted mBHI medium (Fig. 2A). In one case, the maximum OD of the consumer was even 10% higher than that in undiluted mBHI medium, i.e., when B. caecimuris was grown in *A. muciniphila* spent medium (Fig. 2A). More generally, six out of the eight consortium members grew to nearly the same density as that in pure mBHI medium on *A. muciniphila*’s spent medium, suggesting that *A. muciniphila* makes breakdown products of the complex glycoprotein mucin available to the community. Conversely, *A. muciniphila* grew poorly in most spent media except those of Lactobacillus reuteri and B. coccoides, presumably because these two species consumed less of *A. muciniphila*’s main nutrient source, mucin. L. reuteri does not have the genetic repertoire for mucin degradation, while *B. coccoides* does (Table S1D). Hence, the latter may still degrade mucin in mBHI medium but provide other metabolites to *A. muciniphila*.

While the attained maximum ODs were generally lower than those in undiluted mBHI medium, most of them were higher than the half-maximum OD that one would expect from a 1-to-1 dilution (Fig. 2A), indicating the consumption of additional nutrients from culture supernatants or nonoverlapping nutrient preferences between the two species. Besides the above-mentioned nutritional benefit of *A. muciniphila*’s culture supernatant, a particularly beneficial combination was seen between *F. plautii* and *M. intestinale*. Five cultures reached lower maximum ODs than expected from a 1-to-1 dilution, and seven cultures did not grow at all, mostly *M. intestinale*, suggesting either competition for essential nutrients or secretion of inhibitory metabolites. Investigating the OMM species in pure spent media, a parallel study found mainly growth inhibition (20), possibly as a consequence of nutritional competition. The high frequency of positive interactions in our experiments was probably caused by mixing spent medium 1 to 1 with undiluted mBHI medium, which avoids growth inhibition through the exhaustion of essential metabolites.

Cross-fed metabolites were identified from dynamic patterns of extracellular metabolites during growth in the spent medium experiments by FIA-TOFMS. Similar to fresh mBHI medium, 216 annotated metabolites exhibited changing time profiles across all experiments. In total, 76 metabolites were secreted in fresh mBHI medium and consumed in spent media, 31 of which were already hypothesized to be cross-fed in the experiments with fresh mBHI medium (Fig. 1C; Table S2A), providing evidence for 142 metabolic cross-feeding interactions (Fig. 2B; Table S2B). For example, the organic acid succinate was cross-fed seven times as it was produced by several members (Fig. 1C; Table S2B). The largest number (21) of metabolic interactions was observed for C. clostridioforme when grown in the spent medium of *B. caecimuris* although without an apparent effect on the maximum OD (Fig. 2A). With up to 57 consumed metabolites, C. clostridioforme was the most promiscuous species, and *M. intestinale*, at the other extreme, did not consume any of the detected metabolites that were secreted by other consortium members (Fig. 2B). The small growth improvement of *F. plautii* in *M. intestinale*’s medium in the absence of detected cross-feeding suggests either the presence of a not-detected metabolic interaction or the existence of an advantageous nonmetabolic interaction. For a more systematic scoring of consumers and producers, we determined the ratio of consumption to secretion interactions. Representing more than 50% of the OMM consortium in the mouse cecum and colon (Fig. S3) (15), the two *Bacteroidetes* members *B. caecimuris* and *M. intestinale* had the lowest consumption-to-secretion ratio. The second most abundant *in vivo Firmicutes* species, *F. plautii*, had the third-lowest ratio (Table S2C). Thus, *in vivo* abundant OMM members appear to have mainly a provider role within the cross-feeding network through a wide range of secreted compounds. The number of consumed metabolites was highly correlated with genome size (Pearson correlation coefficient of 0.70) and even more highly when considering only cross-fed metabolites (Pearson correlation coefficient of 0.80), consistent with previous observations that specialist bacteria have smaller genomes than generalist bacteria (33, 34) (Table S2C).

10.1128/msystems.01484-21.3 FIG S3 Relative abundance of OMM members in mice colonized with the OMM consortium. Relative abundances in three different locations were determined by metagenomics sequencing. Data were taken from a study reported previously by Yilmaz et al. (21). Download FIG S3, EPS file, 1.7 MB. Copyright © 2022 Pérez Escriva et al. 2022 Pérez Escriva et al. https://creativecommons.org/licenses/by/4.0/ This is an open-access article distributed under the terms of the Creative Commons Attribution 4.0 International license.

The 76 cross-fed metabolites include 6 carboxylic acids, 6 amino acids or derivatives thereof, 7 nucleic acids, and 3 carbohydrates (Table S2D). These metabolites may be either a private or a public good, i.e., consumed by two or more members, respectively (Fig. 2C). As typical fermentation end products, carboxylic acids were significantly enriched among the public goods (*P* = 0.001 by a Fisher exact test). For example, the organic acid succinate was cross-fed seven times. Of special interest for anaerobic respiration are malate and fumarate that are secreted by *B. caecimuris* and *C. innocuum*. Malate can be hydrated to fumarate, which, in turn, can function as an electron acceptor to form succinate in anaerobic environments (35, 36). These public goods were consumed by L. reuteri, E. faecalis, *A. muciniphila*, and C. clostridioforme. The presence of several consumers for electron acceptors is potentially relevant for colonization resistance to Salmonella infections, which has been shown to require them during initial gut colonization (36). As potential nitrogen or carbon sources (29), amino acids were predominant among the private goods. For example, histidine was consumed only by *B. coccoides*, the genome of which encodes a degradation pathway that produces glutamate and formate from histidine. Thus, histidine could be used by *B. coccoides* as a biomass precursor, as a nitrogen source, or to produce formate as an electron donor (37). Another private-good example is cysteine provision by several members to C. clostridioforme (Table S2A). Overall, we thus provide evidence for a dense network of 142 cross-feeding interactions between 10 OMM members (Fig. 2B), mainly through carboxylic acids, amino acids, and nucleobases by the three providers *B. caecimuris*, *M. intestinale*, and *F. plautii*.

**FIG 3:**
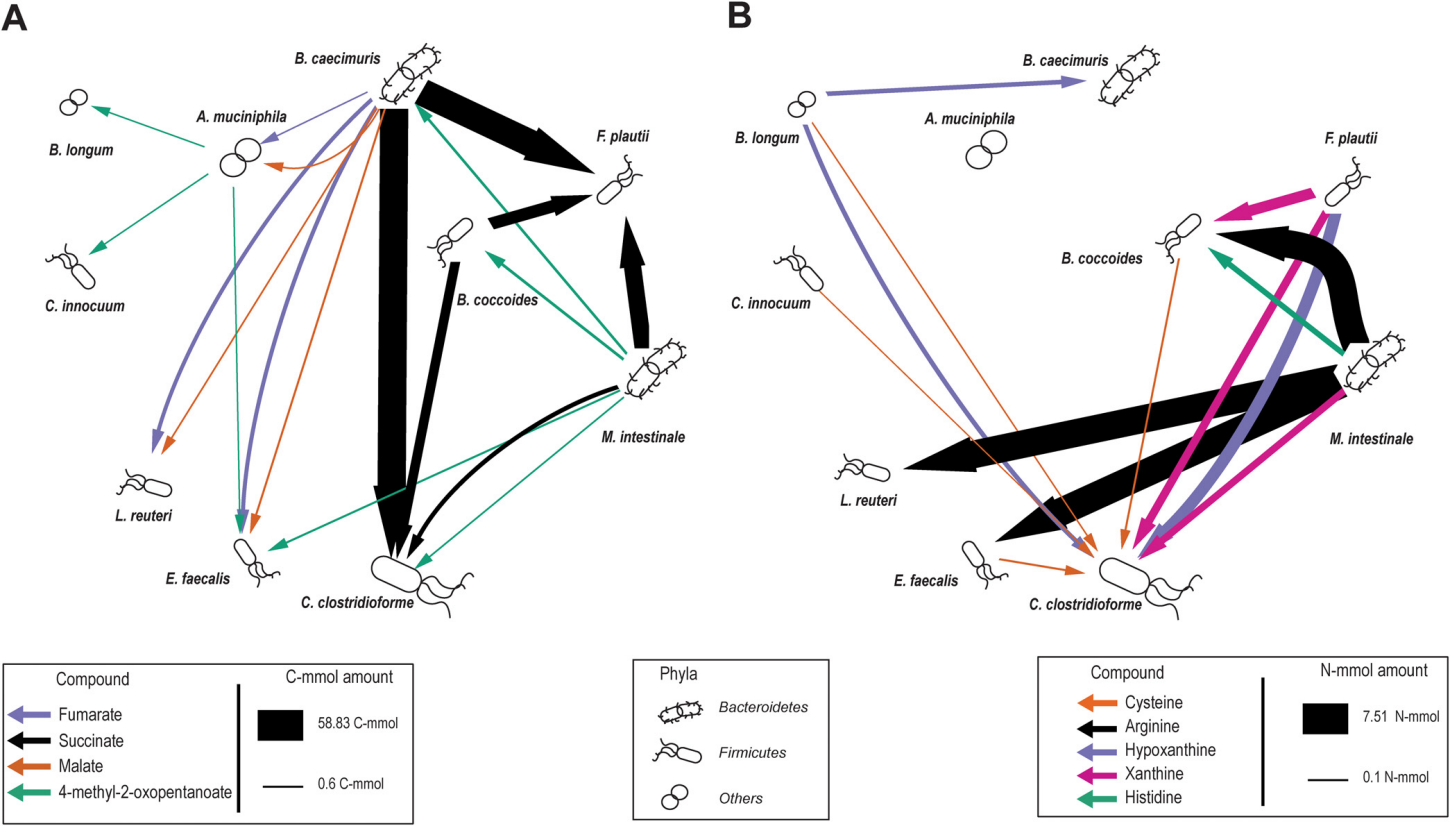
Carbon and nitrogen interaction networks of the OMM consortium. Interactions were inferred from growth experiments in a mix of 50% complex mBHI medium and 50% spent medium of each OMM species. (A) Compound-specific OMM carbon interaction network. (B) Compound-specific OMM nitrogen interaction network. For both networks, the amount of cross-fed compounds containing carbon or nitrogen was quantified and multiplied by the number of carbon or nitrogen atoms per molecule, respectively. Only compounds above 0.1 C-mmol or N-mmol exchange are displayed. Bacteria that represent more than 10% of the community are shown in a larger size in a study by Yilmaz et al. (21).

To assess the relevance of the so-far-mapped interaction network and to unravel the underlying metabolism, we next quantified absolute metabolite concentrations in the above-described spent and fresh medium experiments using a targeted LC-MS method covering 15 of the cross-fed compounds and additional compounds that were expected to be cross-fed from the behavior in fresh media (Fig. 1C). Concentrations of secreted compounds ranged from low micromolar to millimolar and were generally higher for public goods. As expected, fermentation end products accumulated to high concentrations, with succinate being by far the most abundant cross-fed metabolite, reaching 14.7 ± 0.5 mM in *B. caecimuris* spent medium. As another public good, the nucleobases xanthine and hypoxanthine were secreted up to about 1 mM. Among the private goods, the metabolite cross-fed at the highest concentration was histidine (∼0.56 mM) (Fig. 3).

When calculating the C- and N-mol mass balance of cross-fed metabolites from the quantified consumption and production profiles, two rather distinct food webs emerge for carbon and nitrogen (Fig. 3). While *Bacteroidetes* were the main providers of C compounds, with succinate accounting for the major carbon flow (Fig. 3A), species from different phyla contributed to N flow, including *F. plautii*, B. longum, and *M. intestinale*, the latter providing the major N flow through arginine (Fig. 3B). Of note, several amino acids increased over time during the growth of *M. intestinale* (Fig. S2), most likely derived from peptide digestion. Somewhat surprisingly, only arginine and histidine were found to be consumed by other species. Although many metabolites are cross-fed (Fig. 2B), the interaction between any two members was dominated by single C- and N-containing metabolites. The major mass flow of C was mediated by succinate and, to a lesser extent, by malate and fumarate (Fig. 3A), and that of N was mediated by arginine followed by the purine degradation products hypoxanthine and xanthine and histidine (Fig. 3B). Arginine catabolism was previously reported for lactic acid bacteria (38) such as L. reuteri and E. faecalis via the 3-step arginine deaminase system for energy generation with ornithine as a side product (38). Both L. reuteri and E. faecalis contain all necessary genes, including the arginine-ornithine antiporter ArcD that couples arginine uptake to ornithine secretion (Table S3A and B). Consistently, we observed ornithine secretion in both L. reuteri and E. faecalis in mBHI medium (Table S3C) and that this secretion was greater when grown in the spent medium of the arginine-producing species *M. intestinale*. This cross-feeding interaction might also be relevant for the host because ornithine production by *Lactobacillus* has been shown to contribute to the maintenance of a healthy gut mucosa (39).

10.1128/msystems.01484-21.9 TABLE S3 (A) L. reuteri BLAST analysis against the arginine deaminase system proteins. (B) E. faecalis KB1 BLAST analysis against the arginine deaminase system proteins. (C) Ornithine intensities in L. reuteri and E. faecalis in fresh and spent media. Download Table S3, XLSX file, 0.03 MB. Copyright © 2022 Pérez Escriva et al. 2022 Pérez Escriva et al. https://creativecommons.org/licenses/by/4.0/ This is an open-access article distributed under the terms of the Creative Commons Attribution 4.0 International license.

Overall, succinate, malate, and fumarate dominated cross-feeding in the C network, and the amino acids arginine and histidine as well as the nucleobases xanthine and hypoxanthine dominated cross-feeding in the N network. In molar terms, *B. caecimuris* and *M. intestinale* were the predominant providers of C and N, and *F. plautii* was the predominant C consumer and at the same time a relevant N provider. While microbial cross-feeding of succinate has been previously reported in the gut (40), the extent to which malate and fumarate or xanthine and hypoxanthine are cross-fed has not been characterized yet.

### Supplementation reveals physiological benefits of cross-feeding.

**FIG 4:**
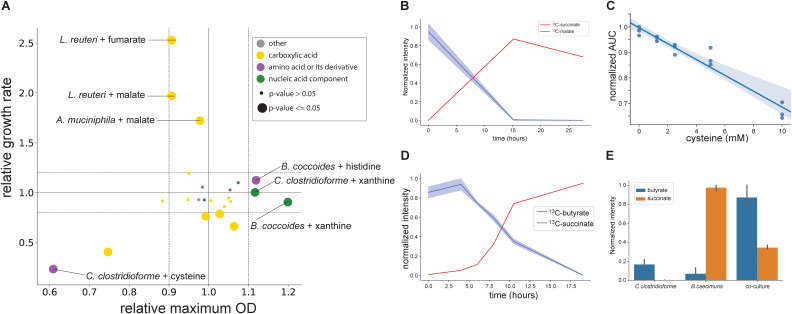
Supplementation and ^13^C-tracing experiments reveal the impact of cross-feeding interactions. (A) Impact of cross-fed nutrient supplementation on the maximum OD and growth rate in mBHI medium supplemented with one metabolite of the indicated compound class (*n* = 3 replicates per experiment). Values are shown relative to the growth rate and maximum OD obtained without supplementation. Color indicates metabolite class, and the dot size is proportional to the significance of the *P* value (by Student’s *t* test). The dotted horizontal lines at 1.1 and 0.9 are shown for reference. (B) Extracellular time course of fully ^13^C-labeled succinate and malate in L. reuteri mBHI medium cultures supplemented with ^13^C-malate. Shaded areas represent the standard deviations from the experiments (*n* = 3 replicates per experiment). (C) Normalized area under the OD curve (AUC) of C. clostridioforme grown in mBHI medium supplemented with different concentrations of cysteine. Shaded areas represent the standard deviations from the means (*n* = 3 replicates per experiment). (D) Levels of ^13^C-succinate and ^13^C-butyrate over time when C. clostridioforme was grown in mBHI medium supplemented with ^13^C-succinate. Shaded areas represent the standard deviations from the means (*n* = 3 replicates per experiment). (E) Levels of succinate and butyrate in monocultures and coculture of C. clostridioforme and *B. caecimuris* in GMM normalized to the maximum value across all experiments (*n* = 3 replicates per experiment).

With nine compounds being cross-fed at >100 μM (Table S4A), we next investigated the physiological relevance of this C and N flux between species. Cultures of consuming species were grown in mBHI medium separately supplemented with 10 mM (each) the 9 compounds to determine the specific growth rate and maximum OD. While most supplemented metabolites did not affect either of these physiological parameters, as might be expected in a rich complex medium like mBHI medium, malate almost doubled the growth rates of L. reuteri and *A. muciniphila*, and fumarate had an even more dramatic effect on L. reuteri (Fig. 4A). Supplementation with N-containing compounds affected only the maximum OD, i.e., a 10 to 20% improvement of *B. coccoides* and C. clostridioforme by xanthine and the former also by histidine. Although arginine has been reported as a C and N source (41), supplementation did not improve the growth of L. reuteri or E. faecalis. Supplementation with the amino acid cysteine had a drastic negative impact on C. clostridioforme (Fig. 4A), although it was consumed during growth in spent media (Fig. 3B).

10.1128/msystems.01484-21.10 TABLE S4 (A) Cross-fed metabolite *in vitro* supplementation results. (B) Intracellular metabolites enriched in C. clostridioforme when [^13^C]succinate was provided. Download Table S4, XLSX file, 0.01 MB. Copyright © 2022 Pérez Escriva et al. 2022 Pérez Escriva et al. https://creativecommons.org/licenses/by/4.0/ This is an open-access article distributed under the terms of the Creative Commons Attribution 4.0 International license.

The higher biomass yield is most likely explained by providing energetically expensive N-containing building blocks for biomass; i.e., the synthesis of nucleotides and histidine requires several ATP per molecule. The dicarboxylic acids malate and fumarate could potentially be oxidized to succinate in anaerobic respiration, allowing bacteria to generate more ATP (36). To confirm their metabolic fate, we supplemented L. reuteri and *A. muciniphila* mBHI medium cultures with fully ^13^C-labeled malate. Consistent with this hypothesis, both cultures consumed ^13^C-malate and secreted fully ^13^C-labeled succinate (Fig. 4B; Fig. S5A).

10.1128/msystems.01484-21.5 FIG S5 (A) Time course of extracellular fully ^13^C-labeled succinate and malate in *A. muciniphila* in mBHI medium cultures supplemented with [^13^C]malate. Shaded areas represent the standard deviations from the experiments (*n* = 3 replicates per experiment). (B) Intracellular fully labeled fraction of pyruvate in C. clostridioforme when grown with [^13^C]cysteine or double-distilled water (ddH_2_O) (*n* = 3 replicates per experiment). Download FIG S5, EPS file, 1.5 MB. Copyright © 2022 Pérez Escriva et al. 2022 Pérez Escriva et al. https://creativecommons.org/licenses/by/4.0/ This is an open-access article distributed under the terms of the Creative Commons Attribution 4.0 International license.

To assess the strong negative impact of cysteine, we supplemented C. clostridioforme cultures with fully ^13^C-labeled cysteine and identified pyruvate as the degradation product (Fig. S5B). The inevitable by-product of this reaction is hydrogen sulfide, with an important role in maintaining physiological homeostasis in the gut (42) but toxic for the host and bacteria at higher concentrations (43). Indeed, increasing cysteine supplementation augmented the growth inhibition (Fig. 4C), suggesting that hydrogen sulfide was the inhibiting agent. This inhibition might also be relevant *in vivo* because hydrogen sulfide is one of the four main gut microbiome gases (42).

### Succinate cross-feeding is a main source of butyrate production in the OMM consortium.

Since succinate accounted for by far the greatest mass flow of carbon in the OMM cross-feeding network (Fig. 3A) and is a known microbiota-derived metabolite with important roles in gut homeostasis, pathogen susceptibility, and inflammation-related diseases (44), we next investigated its metabolic fate. Under anaerobic conditions, succinate is generally considered a reduced end product (36) or a key intermediate of propionate synthesis by primary fermenters such as *Bacteroides*, when CO_2_ is limiting (44, 45). To elucidate the fate of succinate in one of the two main consumers, C. clostridioforme, we grew cultures in mBHI medium supplemented with fully ^13^C-labeled succinate and analyzed its intracellular metabolome during mid-exponential growth at isotopic steady state by untargeted LC-MS. Among the fully labeled intracellular metabolites, we found several intermediates of butyrate production (Fig. S4A and Table S4B). The conversion of succinate to butyrate has been described for Clostridium kluyveri (46) and, more recently, also for the gut pathogen Clostridioides difficile (47). In the latter case, it was shown to constitute an important metabolic niche in the absence of other succinate consumers after antibiotic treatment (47). While this conversion to butyrate does not produce ATP, it acts as an electron sink, regenerating NAD^+^ from NADH (Fig. S4B). Consistently, we observed the consumption of ^13^C-succinate and the secretion of fully labeled ^13^C-butyrate (Fig. 4D), providing strong evidence for the operation of this pathway in C. clostridioforme.

10.1128/msystems.01484-21.4 FIG S4 (A) Succinate-to-butyrate conversion pathway proposed for C. clostridioforme. Metabolites highlighted in boldface type were detected as being fully labeled intracellularly when C. clostridioforme was supplemented with [^13^C]succinate. (B) Growth of C. clostridioforme in gut microbiota medium with either sorbitol or sorbitol and succinate. Shaded areas represent the standard deviations from the means (*n* = 3). Download FIG S4, EPS file, 1.5 MB. Copyright © 2022 Pérez Escriva et al. 2022 Pérez Escriva et al. https://creativecommons.org/licenses/by/4.0/ This is an open-access article distributed under the terms of the Creative Commons Attribution 4.0 International license.

To verify whether succinate cross-feeding occurs in coculture, C. clostridioforme and *B. caecimuris* were grown in mono- and cocultures. As described above (Fig. 1C and Fig. 3A), monocultures of *B. caecimuris* produced succinate but no butyrate, while C. clostridioforme produced a small amount of butyrate (Fig. 4E). In coculture, however, butyrate accumulated to much higher levels at the expense of succinate, demonstrating succinate cross-feeding and butyrate fermentation in C. clostridioforme. Overall, these results show that the mouse commensal C. clostridioforme is a butyrate producer, and succinate cross-feeding within the OMM consortium might be a relevant source of butyrate.

While this cross-feeding does not provide an *a priori* fitness benefit to C. clostridioforme, butyrate is a host-relevant metabolite that can be used as a carbon source by colonocytes (48) and has anti-inflammatory properties (49). Since succinate consumption improves gut colonization by the pathogen C. difficile in the presence of the dietary sugar sorbitol, which requires NAD^+^ for its catabolism (47), we grew C. clostridioforme in rich gut microbiota medium with sorbitol or succinate and sorbitol as the carbon sources. Akin to C. difficile, succinate availability improved the fitness of C. clostridioforme albeit to only a small extent (Fig. S4C). Thus, we show that the benefits of succinate cross-feeding for C. clostridioforme are context dependent and suggest that it might be a relevant interaction *in vivo* in the presence of the abundant diet-derived carbon source sorbitol.

## DISCUSSION

Our results are based on an experimental approach that combines systematic *in vitro* cultivation in rich and spent media with dynamic exometabolomics to characterize metabolic fingerprints of species and infer potential metabolic interactions in microbial communities. Beyond the identification of producers of well-known gut microbiome fermentation products such as acetate, propionate, butyrate, and lactate (40), our systematic approach mapped 142 interactions in the recently introduced synthetic mouse gut consortium OMM (15). As the major constituents with up to 50% of the consortium in the mouse colon (21), the *Bacteroidetes* phylum representatives *B. caecimuris* and *M. intestinale* were the main providers, in terms of both the numbers of compounds and mass flow. The former dominated the C interaction network primarily with the secretion of vast amounts of succinate but also produced malate and fumarate that were used by several other species. While *M. intestinale* dominated the N interaction network with the secretion of large amounts of arginine, several other species contributed further N-containing compounds such as histidine and the nucleobases hypoxanthine and xanthine. As the member with the largest genome, C. clostridioforme was by far the most promiscuous consumer of metabolites in the consortium. *F. plautii* assumes a special role within the community in being one of the two main consumers of C in the form of succinate and a major producer of N-containing compounds.

While cross-feeding of carboxylic and amino acids was known to occur in the mammalian gut (50), cross-feeding of nucleobases is, to our knowledge, a new observation. The extents of xanthine and hypoxanthine interactions in our consortium and nucleobase secretion by other gut microbes such as Escherichia coli (51) suggest that this cross-feeding might not be limited to the OMM consortium. Such purine metabolites were recently shown to affect host traits, including aging (52), irritable bowel syndrome (53), or the maintenance of mucus barrier function (54). Supplementation experiments with the most abundant cross-fed metabolites demonstrated physiological benefits in several cases, even in rich complex medium. Generally, N cross-feeding improved the biomass formation of *B. coccoides* and C. clostridioforme, in particular through histidine and xanthine. Although cross-fed at large quantities, arginine did not provide any fitness benefit to the consuming species but led to the production of ornithine, a metabolite implied for mucosal health (39) that can also induce the biosynthesis of enterobactins by E. coli during infection (55).

In contrast to the N compounds, cross-feeding with C-containing compounds affected only the rate of biomass formation, presumably by allowing the production of more energy per carbon source. In particular, the anaerobic electron acceptor fumarate and malate, which can be hydrated to fumarate, greatly increased the specific growth rates of L. reuteri and *A. muciniphila*. In particular, the higher growth rate of *A. muciniphila* in the presence of malate might be a relevant interaction *in vivo* since both *A. muciniphila* and *B. caecimuris* are the two most abundant members of the OMM consortium in the cecum and colon of adult mice (21). Alternative electron acceptors like malate and fumarate have been shown to improve the *in vivo* fitness of E. coli (35) and the pathogen Salmonella (56). Their production by *Bacteroides* species has been reported previously (57) and is generally linked to the presence of *Bacteroidetes* (58).

While physiological benefits to the consumer are a strong argument for relevant cross-feeding, a cross-feeding interaction might also be beneficial to the host. An example is succinate, which was reduced by C. clostridioforme to butyrate, a microbiome-derived metabolite shown to impact host physiology as a carbon source, have anti-inflammatory function, or act as a signaling compound (47). Butyrate production from carbohydrates or organic acids has been described extensively (59), in particular the conversion of succinate to butyrate by C. kluyveri (46) and, more recently, also for the gut pathogen C. difficile (47). Through isotopic tracing and coculture experiments with the major OMM succinate producer *B. caecimuris*, we demonstrated that C. clostridioforme also produces butyrate from succinate, which constituted the quantitatively largest cross-feeding flux in our consortium. This cross-feeding might be relevant *in vivo* not only as a host source of butyrate but also for the depletion of the inflammatory succinate (60) produced by other species. While our findings are limited to the OMM consortium, the prevalence of succinate producers such as *Bacteroidetes* in the gut microbiome indicates that succinate cross-feeding to butyrate might be a relevant source of butyrate in the gut microbiome.

The strongest negative fitness effect was seen for cysteine consumption by C. clostridioforme. While cysteine inhibition of amino acid biosynthesis has been reported for E. coli (61), we confirmed its degradation to pyruvate through isotopic tracing. This degradation releases hydrogen sulfide, one of the four relevant microbiome-derived gases (42). The origin of microbiome-derived hydrogen sulfide is often associated with the presence of sulfate-reducing bacteria, but members of this bacterial family are rather infrequent in the human microbiome (62) and absent in the OMM consortium. Given that C. clostridioforme is an abundant OMM member *in vivo* (21), it might play a key role in the degradation of cysteine and the production of hydrogen sulfide in mice harboring the OMM consortium. Consistently, a recent study reported cysteine consumption in two other C. clostridioforme strains (58), suggesting that this species is a relevant cysteine consumer in the gut. Our findings are consistent with the recent notion that beyond dissimilatory hydrogen sulfide formation by sulfate reducers, cysteine catabolism is also ubiquitous and an underestimated source of hydrogen sulfide in the human gut (63).

Comprehensive characterization of the cross-feeding network suggests that the OMM consortium is highly connected at the metabolic level with distinct N and C interaction networks. Quantification and metabolic characterization of the main interactions revealed microbe-microbe interactions but also potential interactions with the host through metabolic end products, including butyrate, hydrogen sulfide, and ornithine. While the *in vitro* experiments with complex mBHI medium and spent medium used here demonstrate only potential metabolic interactions in the gut, the recovery of known interactions, consistency with the expected *in vivo* species abundance, and the demonstration of physiological relevance suggest that many of the cross-feeding interactions may also be relevant *in vivo*. The removal of C. clostridioforme from the OMM consortium, for example, more than halved the *in vivo* concentrations of butyrate (64), suggesting that a major fraction of butyrate formation in OMM mice originates from the here-identified succinate cross-feeding between C. clostridioforme and the OMM consortium’s succinate producers. Likewise, anaerobic respiration is important for Salmonella enterica serovar Typhimurium to colonize OMM mice (64), and our data provide evidence for *B. caecimuris* as a major producer of electron acceptors for anaerobic respiration within the consortium. Moreover, the inferred cross-feeding network can help identify key interactions that are missing within the OMM consortium. For example, cross-feeding of lactate is important in the gut microbiota (59) and contributes to butyrate formation (65). While several members of the OMM consortium produced lactate, no consortium member consumed it. This lack of lactate cross-feeding within the OMM consortium is consistent with the reported higher levels of lactate in the cecum of adult OMM mice than in mice colonized with a specific-pathogen-free microbiome (66). Overall, our findings show that many crucial metabolic features of the gut microbiota are represented within the OMM consortium and strengthen its relevance as a model for the mouse gut microbiota.

## MATERIALS AND METHODS

### Chemicals and strains.

All chemicals were purchased from Sigma-Aldrich. The OMM species were kindly provided by Andrew Macpherson (67).

### Medium preparation.

Modified BHI (mBHI) medium contained 37 g L^−1^ brain heart infusion base, 5 mg L^−1^ hemin, 250 mg L^−1^ cysteine-HCl, 250 mg L^−1^ Na_2_S · 9H_2_O, 0.5 mg L^−1^ menadione, and 0.25 g L^−1^ mucin from porcine stomach type II. Gut microbiota medium was prepared as described previously (68) except with no addition of SCFA and using sorbitol (0.5%, wt/vol) as the only carbon source.

### Fresh and spent medium experiments.

All strains were grown under anoxic conditions in an anaerobic chamber (Coy Laboratory Products Inc., MI, USA) filled with an anaerobic gas mix (5% [vol/vol] carbon dioxide, 5% [vol/vol] hydrogen, 90% [vol/vol] nitrogen) at 37°C. Liquid cultures grown overnight in 10 mL mBHI medium were prepared for each species from frozen stocks. For experiments with fresh mBHI medium, 50 mL mBHI medium was dispensed in 150-mL serum bottles (VWR International and Omnilab AG) sealed with a butyl rubber septum and inoculated from a preculture grown overnight to an initial OD of 0.05. For every consortium member, three to four replicates were incubated at 37°C and stirred at 300 rpm with a small cross-shaped stir bar (2-cm diameter). Aliquots for culture density measurements and metabolomics were withdrawn with a 1-mL syringe and a 23-gauge BD Precisionglide syringe needle through the rubber septum over the entire growth curve to capture lag, exponential, and stationary phases (see Table S1C in the supplemental material).

Spent media were prepared from cultures in stationary phase. For this purpose, culture broth was dispensed in 50-mL Falcon tubes and centrifuged at 3,500 rpm for 10 min at 4°C, and the supernatant was filter sterilized (Polyethersulfone membranes with a 0.22-μm pore size). Aliquots of spent media were stored at −20°C. Individual aliquots were thawed and equilibrated in the anaerobic chamber overnight to remove dissolved oxygen before utilization. For spent medium experiments, Hungate tubes were filled with 10 mL of a 1:1 mixture of mBHI medium and spent medium from the specified species. For every spent medium experiment, Hungate tubes were inoculated in duplicate with 100 μL from an mBHI medium preculture grown overnight in the stationary state to an initial OD of approximately 0.05. The optical density was monitored over the course of the experiment by measuring it directly from the Hungate tube with a Biowave CO8000 cell density meter (VWR International). Similarly, for the fresh medium experiments, samples were collected over the course of the growth curve, trying to capture the different phases of bacterial growth (Table S1C).

### Mass spectrometry analysis.

Aliquots for metabolomics analysis were prepared by centrifugation to separate cells from the culture supernatant and stored at −80°C until further use, as previously described (69). For untargeted analysis, 40-fold-diluted and centrifuged samples were injected into an Agilent 6520 time of flight mass spectrometer operated in negative mode at a 2-GHz extended dynamic range (EDR) and with a mass/charge ratio (*m/z*) range of 50 to 1,000. The mobile phase was 60:40 (vol/vol) isopropanol-water and 1 mM NH_4_F at pH 9.0 for negative mode. For online mass axis correction, mobile phases were supplemented with hexakis(1H,1H,3H-tetrafluoropropoxy)phosphazine and 3-amino-1-propanesulfonic acid for online mass correction. The injection sequence was randomized. Data were acquired in profile mode, centroided, and analyzed with Matlab (Mathworks, Natick, MA). Missing values were filled by recursion in the raw data. Upon the identification of consensus centroids across all samples, ions were putatively annotated by accurate mass and isotopic patterns. Starting from the comprehensive list of bacterial metabolites, a database was compiled by extracting the metabolites present in the KEGG genomes of gut bacteria (70). All formulas matching the measured mass within a mass tolerance of 0.003 Da were enumerated. As this method does not employ chromatographic separation or in-depth MS2 characterization, it is not possible to distinguish between compounds with identical molecular formulas. The confidence of annotation reflects level 4, but in practice, in the case of intermediates of primary metabolism, it is higher because they are the most abundant metabolites in cells.

Short-chain fatty acids were quantified via the 3-nitrophenylhydrazine (3-NPH) derivatization method developed by Han and colleagues (71). Briefly, 40 μL of 10-fold-diluted samples was mixed with 20 μL of a 120 mM 1-ethyl-3-(3-dimethylaminopropyl) carbodiimide-HCl–6% (vol/vol) pyridine solution and 20 μL of a 200 mM 3-NPH–HCl solution. Samples were incubated at 37°C for 30 min and diluted 25 times with 10% aqueous acetonitrile. Finally, samples were centrifuged for 2 min at 3,500 rpm, and the clear supernatant was used for analysis. Samples were measured with the same MS system as the one described above. Chromatographic separation was performed on a 50- by 2.1-mm, 130-Å, 1.7-μm Acquity ultraperformance liquid chromatography (UPLC) ethylene-bridged hybrid (BEH) C_18_ column (Waters) using a mobile phase A containing H_2_O and 0.1% formic acid and a mobile phase B containing acetonitrile and 0.1% formic acid. An injection volume of 2 μL was used, and elution was achieved using the following gradient: initial conditions of 83% mobile phase A at 1,100 μL/min, 0.2 min of 83% A, 1.9 min of 82% A, 2.8 min of 60% A, 3.0 min of 0% A, 3.50 min of 0% A, and 3.51 min of 83% A. Online mass calibration was performed using a second spray needle and a constant flow (5 μL/min) of a reference solution containing purine and hexakis(1H,1H,3H-tetrafluoropropoxy)phosphazine (HP-0921; Agilent Technologies). Compounds were identified based on the retention time of chemical standards and their accurate mass (tolerance of 20 ppm). MassHunter quantitative analysis software (version 7.0; Agilent) was used for peak integration.

Quantitative measurement of selected metabolites was performed by liquid chromatography coupled to MS. Chromatographic separation via hydrophilic interaction liquid chromatography (HILIC) was performed on an AdvanceBio MS spent medium column (50 by 2.1 mm; Agilent Technologies) using a mobile phase A containing H_2_O and 10 mM ammonium acetate (pH 9.0) and a mobile phase B containing acetonitrile and 10 mM ammonium acetate (pH 9.0). Samples were prepared as described above and diluted 5 times in a 50:50 mixture of water-acetonitrile. One microliter of the 100-fold-diluted sample was injected, and elution was achieved using the following gradient: initial conditions of 5% mobile phase A at 1,000 μL/min, 0.25 min of 5% A, 0.75 min of 50% A, 1.0 min of 65% A, 1.25 min of 65% A, 1.26 min of 95% A, and 2.25 min of 95% A. The quadrupole time of flight (qTOF) system (Agilent 6520) was operated in negative mode at a 2-GHz extended dynamic range with an *m/z* range of 50 to 1,000 and the following source parameters: VCap of 3,500 V, nozzle voltage of 2,000 V, gas temperature of 325°C, drying gas at 5 L/min, and a nebulizer at 30 lb/in^2^ gauge. Online mass calibration was performed using a second spray needle and a constant flow (5 μL/min) of a reference solution containing purine and hexakis(1H,1H,3H-tetrafluoropropoxy)phosphazine (HP-0921; Agilent Technologies). Compounds were identified based on the retention time of chemical standards and their accurate mass (tolerance of 20 ppm). MassHunter quantitative analysis software (version 7.0; Agilent) was used for peak integration, and quantification was performed in the software based on a calibration curve of chemical standards.

Cysteine and cystine quantification was performed by liquid chromatography coupled to MS using a 5500 QTrap triple-quadrupole mass spectrometer in positive mode with the multiple-reaction monitoring (MRM) scan type (AB Sciex, Foster City, CA). Separation was performed using a HILIC Plus rapid-resolution high-definition (RRHD) column (1.8 μm, 2.1 by 100 mm; Agilent Technologies) using a mobile phase A containing H_2_O with 0.1% (vol/vol) formic acid and 10 mM ammonium formate and a mobile phase B containing acetonitrile with 0.1% (vol/vol) formic acid. Five microliters of an 80-fold-diluted sample was injected, and elution was achieved using the following gradient: initial conditions of 10% mobile phase A at 400 μL/min, 2.0 min of 60% A, 3.0 min of 60% A, 5.0 min of 10% A, and 6.0 min of 10% A. Data acquisition was performed with Analyst 1.7.1 software (Sciex, Darmstadt, Germany), and peak integration was performed using in-house software. To account for the oxidation over time of cysteine in spent media (72), cysteine was quantified by adding the concentration of cysteine plus 2 times the concentration of cystine.

For labeling experiments, 100-fold-diluted and centrifuged samples were injected into an Agilent 6546 time of flight mass spectrometer operated in negative mode with an *m/z* range of 50 to 1,000. Chromatographic separation was performed on a 30- by 2.1-mm, 1.7-μm Acquity UPLC BEH C_18_ column (Waters) using a mobile phase A containing H_2_O and 0.1% acetic acid and a mobile phase B containing methanol and 0.1% acetic acid. An injection volume of 2 μL was used, and elution was achieved using the following gradient: initial conditions of 83% mobile phase A at 1,100 μL/min, 0.2 min of 83% A, 1.9 min of 82% A, 2.8 min of 60% A, 3.0 min of 0% A, 3.50 min of 0% A, and 3.51 min of 83% A. Online mass calibration was performed using a second spray needle and a constant flow of a reference solution containing purine and hexakis(1H,1H,3H-tetrafluoropropoxy)phosphazine (HP-0921; Agilent Technologies). After processing of raw data as previously described (24), *m/z* features (ions) were annotated by matching them to the accurate mass-to-sum formulas of a comprehensive list of a bacterial metabolite database with a 0.001-Da mass accuracy assuming single deprotonation ([M − H]). Notably, this metabolomics method cannot distinguish between isobaric compounds, e.g., metabolites having identical *m/z* values (e.g., leucine versus isoleucine).

### Identification of consumed and secreted metabolites.

Increasing and decreasing metabolites were identified from the untargeted metabolomics data set as annotated ions with significant correlation to the OD of the bacteria over time (Pearson correlation coefficient of >0.7; *P* value of <0.05) or a significant goodness of linear or exponential fit (*R*^2^ of >0.7; *P* value of <0.05). The latter accounts for metabolites that might be exhausted before the end of the growth experiment or constantly produced throughout the experiment. Furthermore, the maximum fold change between the initial time point and any other point had to be higher than −1.37 for consumed metabolites and higher than 1.20 for a secreted metabolite. These thresholds were determined based on an average fold change observed for every annotated ion in mBHI medium in a dilution series. In brief, 20- to 720-fold dilutions of mBHI medium were measured by FIA-MS in triplicate. Since FIA-MS is sensitive to matrix effects, ion count changes cannot be directly translated into an equivalent metabolite change. To remove background ions that are not derived from mBHI medium, the above-mentioned thresholds were determined, focusing only on annotated metabolites that had a highly significant negative correlation to the dilution factor (Pearson correlation of less than −0.75) (Fig. S6A and B). To determine average fold changes of consumed metabolites, we used measurements of the annotated metabolites in 40- to 80-fold dilutions. To determine average fold changes of secreted metabolites, we used measurements of annotated metabolites in 20- to 40-fold dilutions. Average fold changes were 1.37 and 1.20 for consumed and secreted metabolites, respectively (Fig. S6C).

10.1128/msystems.01484-21.6 FIG S6 (A) Distribution of Pearson correlation coefficients between dilution factors and ion intensities of the metabolites annotated in mBHI medium via FIA-MS. (B) Normalized ion intensity over the dilution factor for metabolites with a Pearson correlation coefficient below −0.75. Shaded areas represent the standard deviations from the means (*n* = 3 replicates per experiment). (C) Average fold changes for annotated ions that had a Pearson correlation coefficient below −0.75 from 20- to 40-fold dilutions (increase) and from 40- to 80-fold dilutions (decrease). Download FIG S6, EPS file, 1.9 MB. Copyright © 2022 Pérez Escriva et al. 2022 Pérez Escriva et al. https://creativecommons.org/licenses/by/4.0/ This is an open-access article distributed under the terms of the Creative Commons Attribution 4.0 International license.

### *In vitro* supplementation experiments.

Liquid cultures grown overnight in 5 mL of mBHI medium were prepared for each species from frozen stocks. Solutions of supplemented metabolites were prepared in deionized water, titrated to pH 7, filter sterilized (0.22 μm), and stored at −20°C. Stock solutions of supplements were thawed and equilibrated overnight in the anaerobic chamber the day before the start of the experiment. Species were then grown anaerobically in triplicates in 10-mL Hungate tubes consisting of 90% (vol/vol) mBHI medium and 10% (vol/vol) supplement at 10 mM. Growth curves were acquired by monitoring the OD with a Biowave CO8000 cell density meter (VWR International). Growth rates were inferred by fitting growth curves to the OD as a function of time with a four-parameter logistic function (73).

### Isotopic tracer experiments.

Liquid cultures grown overnight in 5 mL of mBHI medium were prepared for each species from frozen stocks. Solutions of labeled supplemented metabolites were prepared in deionized water, titrated to pH 7, filter sterilized (0.22 μm), and stored at −20°C. Stock solutions of supplements were thawed and equilibrated overnight in the anaerobic chamber the day before the start of the experiment. Species were then grown anaerobically in triplicates in 10-mL Hungate tubes consisting of 90% (vol/vol) mBHI medium and 10% (vol/vol) supplement at 10 mM except for ^13^C,^15^N-labeled l-cysteine that was supplemented at 1 mM. Growth curves were acquired by monitoring the OD with a Biowave CO8000 cell density meter (VWR International). Supernatant samples were collected and processed as explained above. Intracellular samples were obtained at mid-exponential phase (OD of 0.4 to 0.6) and extracted as previously described (74). In brief, 2-mL aliquots of the cell culture were filtered by vacuum filtration on a 0.45-μm filter. On the filter, cells were washed with 2 mL of prewarmed ammonium carbonate buffer at pH 7.2. Filters with cultured cells were immediately transferred for extraction into a 2:2:1 mixture of an acetonitrile-methanol-water solution at −20°C. Cells were extracted for at least 2 h and centrifuged at 14,000 × *g* at 4°C for 20 min to remove cell debris. The supernatants were dried at 12 Pa and resuspended in 100 μL of deionized water.

### OMM member metabolic potential assessment.

For each of the 10 cultivated strains of the OMM consortium except L. reuteri, E. faecalis, and B. longum, a list of KEGG orthologs (KOs) for the protein-coding genes was obtained from the KEGG genome database (75). For L. reuteri, E. faecalis, and B. longum, the KEGG Automatic Annotation Server (KAAS) with the BLAST default settings (76) was used to obtain a list of KEGG orthologs. Gut metabolic module (GMM) detection was performed as previously described (70). In brief, GMM presence/absence was identified with a detection threshold of >50% coverage.

### Data availability.

Raw mass spectrometry data and growth physiology data can be downloaded from the BioStudies database (https://www.ebi.ac.uk/biostudies/) under accession number S-BSST686.
