# Supplementary figures and images for: Distinct N and C Cross-Feeding Networks in a Synthetic Mouse Gut Consortium
Source: mSystems. 2022 Mar 31;7(2):e01484-21. doi: 10.1128/msystems.01484-21 (PMC9040589; doi:10.1128/msystems.01484-21)

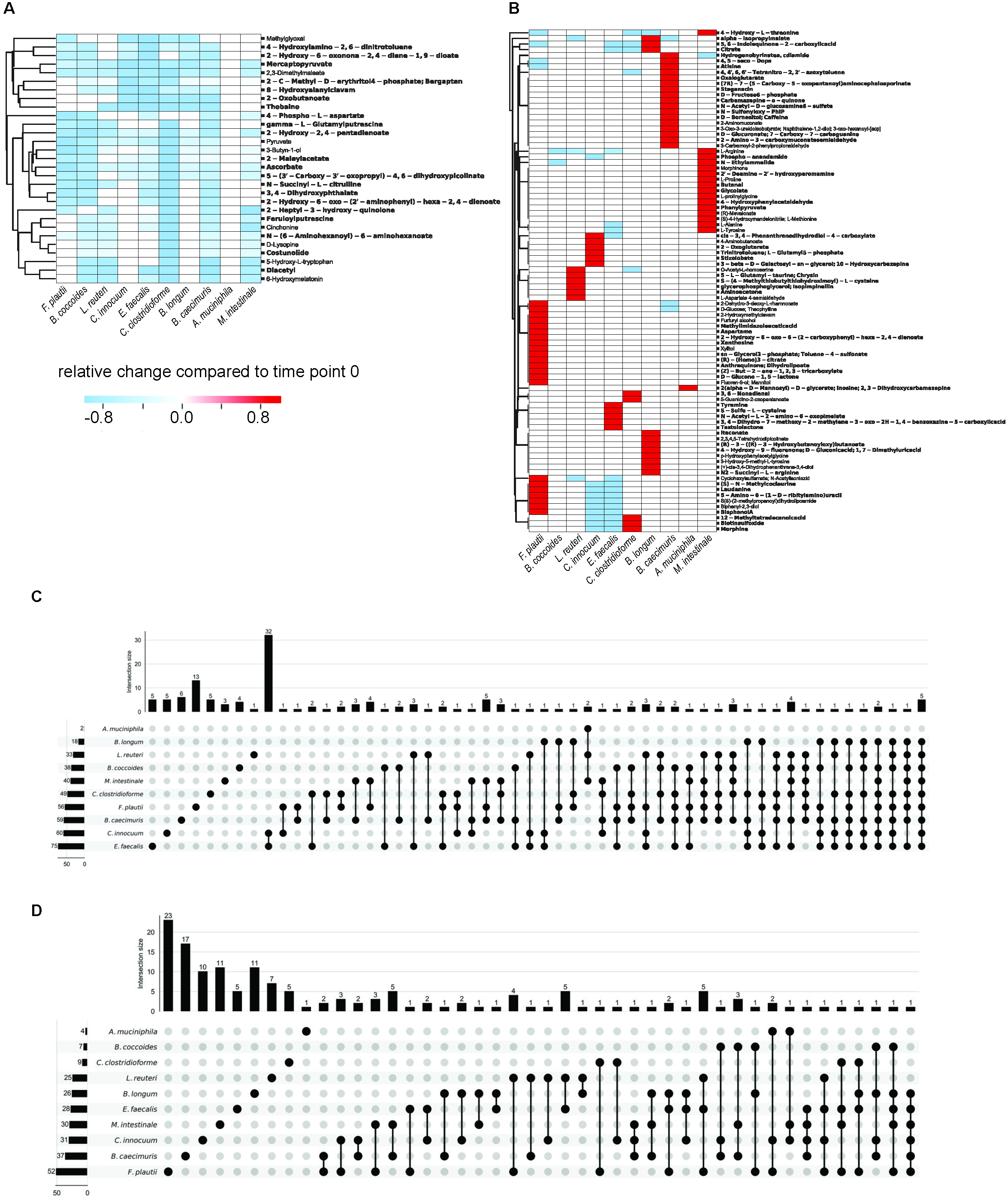

Supplement: FIG S1 [file msystems.01484-21-s0005.tif]
